# Atypical cerebral and cerebellar language organisation: a case study

**DOI:** 10.1186/s40673-015-0036-9

**Published:** 2015-12-10

**Authors:** Kim van Dun, Elke De Witte, Wendy Van Daele, Wim Van Hecke, Mario Manto, Peter Mariën

**Affiliations:** Clinical and Experimental Neurolinguistics, CLIN, Vrije Universiteit Brussel, Brussels, Belgium; Department of Neurology and Memory Clinic, ZNA Middelheim General Hospital, Lindendreef 1, B-2020 Antwerp, Belgium; icoMetrix, Tervuursesteenweg 244, B-3001 Leuven, Belgium; Unité d’Étude du Mouvement, FNRS Neurologie, ULB Erasme, Brussels, Belgium

**Keywords:** Cerebellum, Anterior insula, Schmahmann’s syndrome, Bilateral language representation, CVA, Crossed cerebello-cerebral diaschisis

## Abstract

**Background:**

In the majority of right-handed subjects, language processing is subserved by a close interplay between the left cerebral hemisphere and right cerebellum. Within this network, the dominant fronto-insular region and the contralateral posterior cerebellum are crucially implicated in oral language production.

**Case Presentation:**

We report atypical anatomoclinical findings in a right-handed patient with an extensive right cerebellar infarction and an older left fronto-insular stroke. Standardised neurolinguistic and neurocognitive test batteries were performed. In addition, fMRI, DTI, and SPECT results are reported.

In this patient, disruption of the cerebellocerebral language network due to vascular damage in the left fronto-insular region and right posterior inferior cerebellar artery (PICA) territory did not induce any speech or language deficits. By contrast, executive and behavioural disturbances were found after the right cerebellar stroke. Evidence from fMRI and DTI suggests atypical bilateral language representation (Laterality Index = +0,11). At the cerebellar level, fMRI showed more activated voxels in the left than in the right hemisphere (Laterality Index = +0,66).

**Conclusion:**

We hypothesise congenital bilateral language representation in this patient which might be more advantageous than a typically lateralised distribution of linguistic functions to compensate acute damage to critical language regions. The more activated left cerebellum possibly compensated the functional loss in the right cerebellum after acute damage due to bilateral organisation of language function. However, more research is needed to confirm this hypothesis.

## Background

In a classical language model, expressive and receptive language are primarily subserved by Broca’s area in the left inferior frontal gyrus (BA 44–45) and Wernicke’s area in the left superior and middle temporal gyrus (BA 21–22), which are connected by the arcuate fascicle. Classical theories posit that Broca’s area is responsible for language production, while Wernicke’s area is assumed to subserve speech comprehension [1].

In recent years, however, this traditional topological model of language has been revised and extended. Dronkers [2], for example, identified the left precentral gyrus of the insula as the coordinator of motor speech planning. Using the Activation Likelihood Estimation (ALE) method, Oh et al. [3] assessed the activation of the insular region in 42 fMRI studies, which used language and speech tasks. It was shown that both speech and language consistently activated the left insular region, regardless of which task was used. Ardila et al. [4] confirmed involvement of the left insula in multiple language functions and also pointed out its role in Broca’s aphasia, apraxia of speech, conduction aphasia, and Wernicke’s aphasia.

Recently it has also been shown that the cerebellum plays a crucial role in language processing through a network of cerebello-cerebral pathways [5, 6]. Anatomically, the cerebellum is reciprocally connected to different cerebral association areas via crossed cortico-ponto-cerebellar and cerebello-thalamo-cortical loops. The right cerebellum is linked to the left cerebral hemisphere and the left cerebellum to the right hemisphere [7, 8]. Many studies have found involvement of the cerebellum in a variety of cognitive and affective functions, including language [9–11]. By means of Single-Photon Emission Computed Tomography (SPECT) and Diffusion Weighted Imaging (DWI), several neuroimaging studies identified crossed cerebello-cerebral diaschisis as a possible pathophysiological mechanism to explain cerebellar induced cognitive and affective disorders [9, 10, 12–15].

Schmahmann and Sherman [16] introduced the concept “Cerebellar Cognitive Affective Syndrome” (CCAS) to describe a complex of cerebellar induced cognitive and affective changes. CCAS or Schmahmann’s syndrome [17] comprises a constellation of deficits affecting executive, linguistic, spatial, and affective functions. Since the introduction of Schmahmann’s syndrome, crucial involvement of the cerebellum has been identified in several distinct linguistic processes. Language deficits including agrammatism, anomia, apraxia of speech, apraxic agraphia, dyslexia, agraphia, and even aphasia-like phenomena have been observed after acute cerebellar damage [6]. Neuroanatomical studies investigating the connections of the cerebellum with the supratentorial motor, paralimbic, and association cortices, have shown that the human cerebellum is not only somatotopically organised for motor control but also for higher-order cognitive and affective functions [18]. Typically, a lateralised involvement of the right cerebellar hemisphere is observed in non-motor linguistic processes [6], while the left cerebellar hemisphere is involved in the modulation of typical non-dominant hemisphere functions. This observation has led to the hypothesis of a “lateralised linguistic cerebellum”, subserved by crossed cerebello-cerebral connections [19].

In the vast majority of right-handers (>97 %), and in most left-handers (>70 %), the left hemisphere and right cerebellum subserve language [20, 21]. Right cerebellar lesions can therefore disrupt language processing through crossed cerebello-cerebral networks. In some cases, however, language dominance is atypically located in the right cerebral hemisphere. Atypical right cerebral dominance for language as a maturational variant is a very rare phenomenon [21]. The non-dominant right hemisphere, however, is often crucially involved in the reorganisation of language functions following extensive damage to the language dominant left hemisphere [22, 23]. Brain plasticity mechanisms allow for reorganisation of cognitive functions after cerebral damage. As such, contralateral right hemisphere regions may (partly) take over language functions of the damaged left hemisphere [22]. Brain plasticity is not only operational in the infant brain [24], it can also compensate functional loss due to tumour growth [25] or after a vascular lesion at an adult age [26, 27].

Jansen et al. [28] showed that the cerebellar hemisphere contralateral to the language-dominant cerebral hemisphere is concomitantly activated in language tasks. They studied 14 healthy volunteers. Seven of them displayed atypical right-hemisphere language dominance, the other seven displayed typical left-hemisphere language dominance. Functional Magnetic Resonance Imaging (fMRI) was performed during a letter-cued word generation task. The study showed a consistent pattern of crossed cerebello-cerebral activations during the word generation task. In each subject the cerebellar hemisphere contralateral to the language-dominant cerebral hemisphere was consistently activated [28].

Language is subserved by an extensive network of cerebral and cerebellar areas, connected via subcortical pathways. Typically, left cerebral hemisphere language dominance is observed with right cerebellar involvement through crossed cerebello-cerebral connections. We present the clinical, neurolinguistic and neuroradiological findings of a right-handed patient who did not show any language deficits irrespectively of an old vascular lesion affecting the left anterior insular region and a recent right ischemic stroke affecting the posterior lobe of the cerebellum.

## Case presentation

**Fig. 1:**
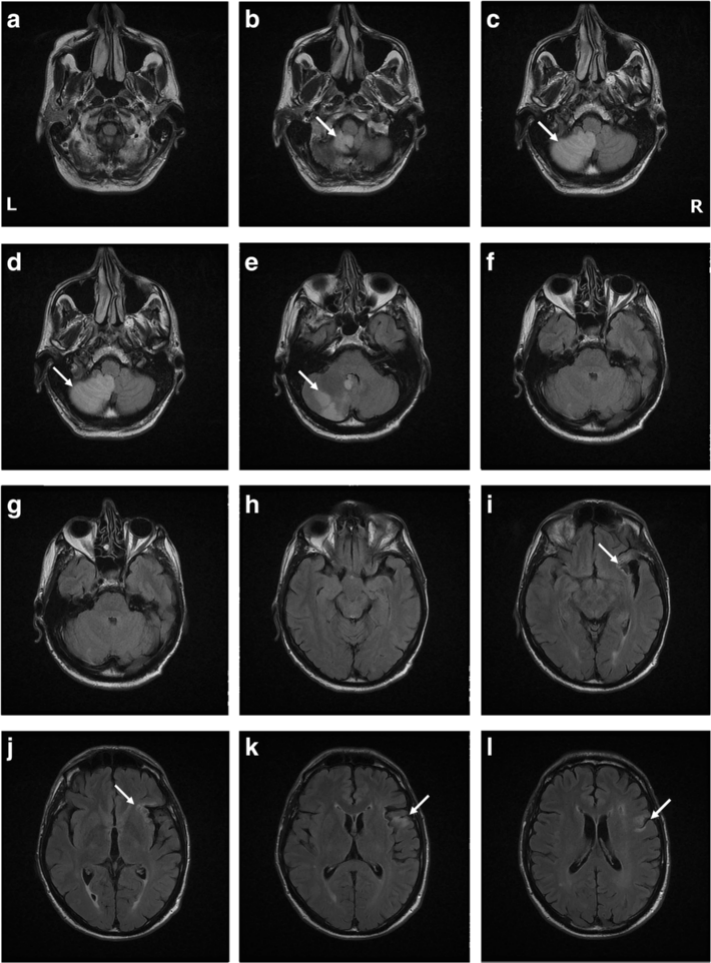
MRI of the cerebellum showing the new cerebellar infarct (**a**-**g**) and the old insular lesion (**h**-**l**) indicated with arrows

A 62-year-old native Dutch-speaking right-handed man was admitted to hospital after an acute episode of balance problems, vertigo, and fainting. On admission one day later he indicated that the room was turning upside down and that he needed support to stand. There was no diplopia. Clinical neurological examination on admission was normal. There was no nystagmus or coordination disturbance. Motor and sensory functions were also normal. Medical history consisted of a cardiac infarction, endocarditis and left femorofibular bypass surgery 10 years before admission. Growth and developmental milestones were normal. The patient had an educational level of 12 years and worked as a trader. He smoked 5 cigarettes a day. Computerised tomography (CT) of the brain showed a hypodense area in the right cerebellar hemisphere, suggestive of ischemic stroke. Subsequently, DWI and T2-weighted FLAIR imaging (MRI) of the brain (Fig. 1) showed a hyperintense lesion in the vascular territory of the right posterior inferior cerebellar artery (PICA), consistent with an acute ischemic stroke. In addition, an old vascular lesion was found in the left anterior insular region extending into the frontal operculum and the precentral gyrus. However, no clinical symptoms were (hetero)anamnestically reported.

### Neuropsychological investigation

In-depth neuropsychological investigations were performed one week after stroke.

A strong and consistent right hand preference was objectified by means of the Edinburgh Handedness Inventory [29] (laterality quotient of +100). Neuropsychological assessments consisted of the revised version of the Wechsler Memory Scale (WMS-R [30]), the Wechsler Adult Intelligence Scale, third edition (WAIS-III [31]), the Hierarchic Dementia Scale (HDS [32]), the Mini Mental State Examination (MMSE [33]), Raven’s Progressive Matrices [34], the Stroop Colour Word Test [35], the Wisconsin Card Sorting Test (WCST [36]), the d2 test of attention [37], the Trail Making Test (TMT [38]) and the Middelheim Frontality Scale (MFS) [39]. The results of these tests are shown in Table 1. A standard deviation (SD) of −1.5 is considered clinically abnormal. A deviation of more than −2 SDs (SD ≤ −2) is considered pathological. Language was assessed with the Aachen Aphasia Test (AAT [40]), the Boston Naming Test-NL (BNT [41]), and verbal fluency tasks. No bilingual tests were performed since the patient as a late learner of French and English did not use these languages on a regular basis. Results of the BNT and the verbal fluency tasks are shown in Table 1 and the results of the AAT in Table 2.

**Table 1:** Neurocognitive test results

Neurocognitive tests	Subject’s (SS/max) RS/max [±SD]	Population Mean / SD
Mini Mental State Examination	(28/30)	28 / 1.8
General cognition		
Wechsler Adult Intelligence Scale-III		
Total IQ (TIQ)	(87) [−0.9]	100 / 15.0
Verbal IQ (VIQ)	(91) [−0.6]	100 / 15.0
- Vocabulary	(9/20) [−0.3]	10 / 3.0
- Similarities	(7/20) [−1.0]	10 / 3.0
- Arithmetic	(7/20) [−1.0]	10 / 3.0
- Digit Span	(7/20) [−1.0]	10 / 3.0
- Information	(12/20) [0.7]	10 / 3.0
- Comprehension	(9/20) [−0.3]	10 / 3.0
Performance IQ (PIQ)	(85) [−1.0]	100 / 15.0
- Digit Symbol Substitution	(6/20) [−1.3]	10 / 3.0
- Picture Completion	(11/20) [0.3]	10 / 3.0
- Block Design	(6/20) [−1.3]	10 / 3.0
- Picture Arrangement	(8/20) [−0.7]	10 / 3.0
- Symbol Search	(6/20) [−1.3]	10 / 3.0
- Matrix Reasoning	(8/20) [−0.7]	10 / 3.0
MEMORY		
Wechsler Memory Scale-Revised		
Verbal Memory Index	(82) [−1.2]	100 / 15.0
Visual Memory Index	(110) [0.7]	100 / 15.0
General Memory Index	(91) [−0.6]	100 / 15.0
Recent Memory Index	(95) [−0.3]	100 / 15.0
Cognitive domains		
Hierarchic Dementia Scale	200/200	
- Orienting	10/10 [0]	10 / 0
- Prefrontal	10/10 [0]	10 / 0
- Ideomotor	10/10 [0.26]	9.94 / 0.23
- Looking	10/10 [0]	10 / 0
- Ideational	10/10 [0.35]	9.97 / 0.17
- Denomination	10/10 [0.35]	9.97 / 0.17
- Comprehension	10/10 [0.35]	9.97 / 0.17
- Registration	10/10 [0.4]	9.86 / 0.35
- Gnosis	10/10 [0.22]	9.92 / 0.37
- Reading	10/10 [0]	10 / 0
- Orientation	10/10 [0]	10 / 0
- Construction	10/10 [0]	10 / 0
- Concentration	10/10 [0.6]	9.69 / 0.52
- Calculation	10/10 [0.26]	9.94 / 0.23
- Drawing	10/10 [0.37]	9.81 / 0.52
- Motor	10/10 [0.4]	9.58 / 1.05
- Remote memory	10/10 [0]	10 / 0
- Writing	10/10 [0.05]	9.94 / 1.26
- Similarities	10/10 [0.4]	9.72 / 0.7
- Recent memory	10/10 [0.57]	9.5 / 0.88
LANGUAGE		
Boston Naming Test^a^	49/60 [−1.9]	55.0 / 3.21
Verbal Fluency		
Semantic categories: total	63 [0.6]	57.1 / 10.06
- animals (1 min)	21	
- transportation (1 min)	10	
- vegetables (1 min)	15	
- clothing (1 min)	17	
Attention/executive function		
WMS-R Attention Index^b^	(60) [−2.7]	100 / 15.0
D2-visuo-motor attention		
- speed^b^	213 [−2.7]	
- errors	14 [0.4]	
- speed/accuracy^b^	199 [−2.7]	
Stroop Colour-Word Test		
- Card I	48″ pc 50	48″
- Card II^b^	79″ pc 10	63″
- Card III^b^	130″ pc 10	99″
Trail Making Test		
- Part A	52″ pc <45	48″
- Part B^b^	227″ pc <10	119″
Wisconsin Card Sorting		
- Number of categories completed	2/128	>4 categories
Raven’s Progressive Matrices	125 [1.7]	100 / 15.0
Middelheim Frontality Scale^b^	7	cut-off ≥ 5

*Max* maximum possible, *SS* standard score, *RS* raw score, *pc* percentile, *SD* standard deviation, *WAIS-III* Wechsler Adult Intelligence Scale 3rd Edition, *TIQ* total intelligence quotient, *VIQ* verbal intelligence quotient, *PIQ* performance intelligence quotient, *WMS-R* Wechsler Memory Scale-revised, ^a^ = clinically abnormal (SD ≤ −1.5); ^b^ = pathological (SD ≤ −2 or pc < 10)

**Table 2:** AAT test results

AACHEN aphasia test	Subject’s results (±SD)	Pc	Max	Mean	SD
Comprehension	120	100	120	108.5	10.24
Auditory: words	30	98	30	26.49	3.30
Auditory: sentences	30	98	30	26.79	3.41
Total	60		60	53.28	6.08
Written: words	30	95	30	28.30	2.29
Written: sentences	30	99	30	26.91	3.39
Total	60		60	55.21	4.90
Token test	0 errors	100	50	2.28	2.75
Spontaneous speech					
Communicative behaviour	5		5	4.63	0.54
Articulation and prosody	5		5	4.63	0.67
Automatisms	5		5	4.59	0.65
Semantic structure	5		5	4.59	0.53
Fonematic structure	5		5	4.54	0.56
Syntactic structure	5		5	4.41	0.55
Imposed Speech					
Total repetition	145	95	150	144.1	8.07
Phonemes	30	88	30	28.91	2.09
Monosyllabic words	30	93	30	29.22	1.32
Loan- & foreign words	30	95	30	28.94	2.31
Compounds	30	99	30	28.45	2.22
Sentences	25 (−1.9)	81	30	28.55	1.90
Total naming	120	100	120	109.3	8.42
Simple nouns	30	97	30	27.92	2.90
Colour names	30	98	30	27.69	1.99
Compounds	30	99	30	28.04	2.61
Sentences	30	100	30	25.69	3.72
Written Language	90	100	90	85.52	7.63
Reading aloud	30	96	30	28.95	1.93
Composing	30	98	30	28.57	2.75
Dictational writing	30	99	30	28	3.67

*SD* standard deviation, *Pc* percentile, *Max* maximum possible, *Mean* population mean

General cognitive screening was normal (MMSE: 28/30; SD: 0,00). The WAIS-III showed a normal total IQ of 87 (SD: −0,87) with a consistent distribution of the verbal (VIQ = 91; SD: −0,60) and performal level (PIQ = 85; SD: −1,00). Table 1 shows a normal distribution of the subtestscores as well. The WMS-R revealed a significant discrepancy of 28 index points between a normal visual (=110; SD: +0,67) and depressed verbal (=82; SD: −1,20) memory index. General memory (=91; SD: −0,60) and recent memory (=95; SD: −0,33) were also within normal range. The HDS yielded normal results. As demonstrated by a pathological WMS-R attention index of 60 (SD: −2,67), general attentional skills were disrupted. The visuomotor d2-test indicated a pathologically slow speed (speed: 213; SD: −2.73). Frontal planning and problem solving were distorted as well. The WCST showed impaired cognitive flexibility and frontal problem solving (2 categories within 128 trials). He performed within the defective range on the TMT part B (pc <10) and the Stroop Colour Word test (pc 10) showed a low average ability to inhibit a competing and more automatic response.

The AAT showed normal spontaneous (maximum score) and imposed speech (repetition: pc 95; naming: pc 100). Oral and written comprehension (pc 100) and reading and writing (pc 100) were also well within the normal range. No clinical evidence was found for dysarthria, apraxia of speech, or aphasia. He obtained a borderline score for visual confrontation naming (BNT: 49/60; SD: −1,87). Semantic verbal fluency, assessed by means of one-minute oral production of words belonging to a specific semantic category, was within the normal range (63 items; SD: +0,59).

At the behavioural level frontal-like behavioural disturbances were noted, as confirmed by a score of 7 on the MFS. The patient behaved in a childish manner and expressed blunt opinions in a theatrical way. Responses to external stimuli were generally characterised by disinhibited behaviour, manifesting as inappropriate behavioural and emotional reactions, overfamiliarity, or flamboyant and impulsive actions. There was a loss of insight and judgment as well as restlessness. Memory and spatial abilities were spared.

### Functional Neuroimaging with SPECT, fMRI, and DTI

#### Acquisition

A quantified Tc-99m-ECD SPECT study was carried out one week poststroke. 740 MBq (20 mCi) Tc-99m-ECD was administered to the patient by means of a previously fixed butterfly needle while he was sitting in a quiet and dimmed room, eyes open and ears unplugged. Acquisition was started 40 min after injection using a three-headed rotating gamma camera system (Triad 88; Trionix Research Laboratory, Twinsburg, Ohio, USA) equipped with lead super-fine fanbeam collimators with a system resolution of 7.3 mm FWHM (rotating radius 13 cm). Projection data were accumulated in a 128 × 64 matrix, pixel size 3.56 mm, 15 s/angle, 120 angles for each detector (3° steps, 360° rotation). Projection images were rebinned to parallel data, smoothed and reconstructed in a 64 × 64 matrix, using a Butterworth filter with a high cut frequency of 0.7 cycles/cm and a roll-off of 5. No attenuation or scatter correction was performed. Trans-axial images with a pixel size of 3.56 mm were anatomically standardised using SPM and compared to a standard normal and SD image obtained from ECD perfusion studies in a group of 15 normally educated healthy adults consisting of eight men and seven women with an age ranging from 45 to 70 years. This normal image was created by co-registration of each normal study to the SPECT template image of SPM using the “normalise” function in SPM. At the same time, the global brain uptake of each study was normalised. On the mean image, 31 ROI’s were drawn and a 31 ROI template was created. Using the normalised studies and the 31 ROI template, the mean normal uptake and SD value (=1 Z score) in each ROI was defined. Patient data were normalised using SPM in the same way and the perfusion uptake in each ROI was calculated. From this uptake, the mean uptake and SD value of the normal database, the Z score for each region can be calculated. A regional Z score of >2.0 is considered significant.

To identify language dominance, fMRI and Diffusion Tensor Imaging (DTI) of the brain were conducted six weeks poststroke on a 3 Tesla (T) MRI scanner (Siemens). For the fMRI, a BOLD sensitive T2*- weighted single shot gradient recalled (GR) echo planar imaging (EPI) sequence (TE/TR: 35/3000 ms; field of view (FOV): 224 × 224 mm; matrix size: 64 × 64; flip angle: 80°) was used resulting in voxel dimensions of 3.5 × 3.5 × 3.0 mm (interleaved). Axial DTI was obtained using a single-shot SE-EPI sequence with the following acquisition parameters: TR: 10.4 s; TE: 100 ms; diffusion gradient: 40mT/m; matrix size: 128 × 128; number of slices = 60; voxel size: 2.0 × 2.0 × 2.0 mm^3^; b = 800 s/mm^2^. Diffusion measurements were performed along 64 directions for a robust estimation of fractional anisotropy (FA), tensor orientation, and mean diffusivity (MD). Data sets were analysed on a separate workstation using SPM8 software. Motion correction was performed, functional data were co-registered to the anatomical image, and the anatomical image was subsequently normalised to the Montreal Neurological Institute (MNI) space using a nonaffine transformation. This transformation was then applied to all registered functional images. After smoothing the functional images with an FWHM of 8 mm, contrasts were calculated. Results were corrected with a familywise error rate (FWE) threshold of 0.05.

## Results

**Fig. 2:**
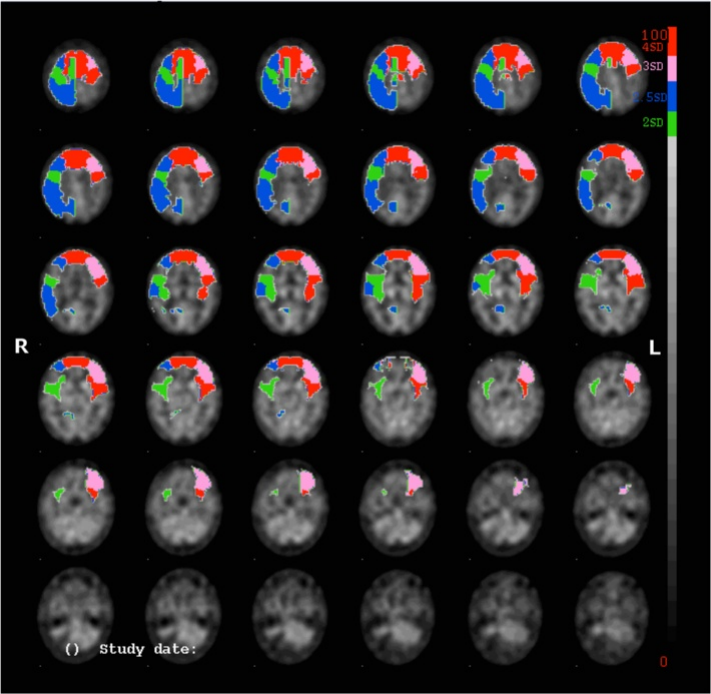
SPECT scan of the brain showing a bilateral hypoperfusion in the prefrontal cortex more pronounced on the left than on the right side. A significant decrease of perfusion is also found in the motor cortex and the right parietal cortex. Left and right are as indicated in the figure

The quantified Tc-99m-ECD SPECT study (Fig. 2) revealed a significant bilateral hypoperfusion in the prefrontal cortex, more pronounced on the left than on the right side (prefrontal lateral: R −2.55 SD; L −3.94 SD; prefrontal medial: R −5.07 SD; L −6.70 SD; prefrontal inferior lateral: R −1.46 SD; L −3.57 SD). A significant hypoperfusion was also found in the motor cortex (R −2.46 SD; L −4.15 SD) and the right parietal cortex (−2.78 SD). Perfusion of the superior cerebellum was normal (R 0.32 SD; L 1.07 SD). The lesion in the inferior cerebellum could not be visualised as the inferior cerebellum was not included in the analysis.

**Fig. 3:**
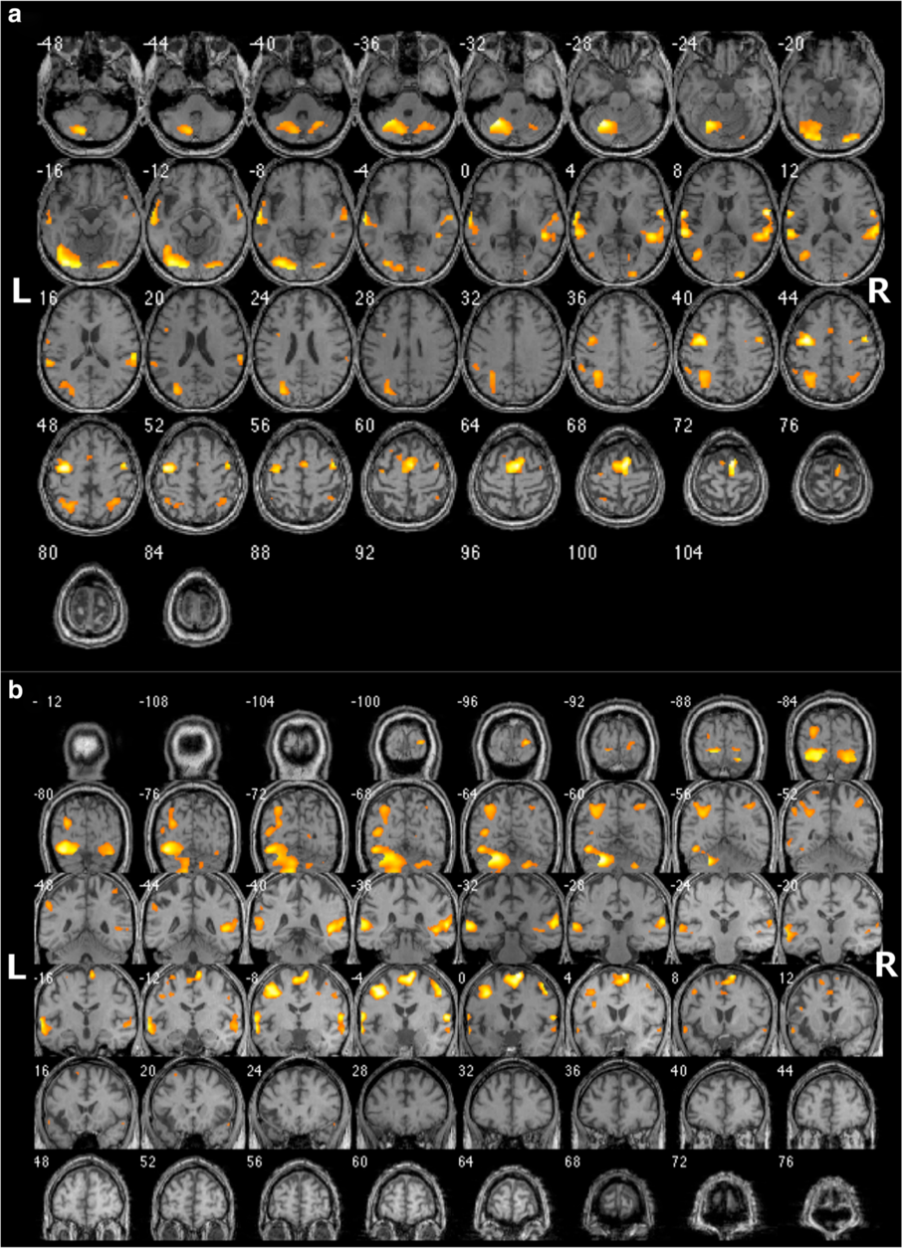
Axial (**a**) and coronal (**b**) fMRI slices during a silent semantic noun-verb association task. Symmetrical language activation is shown, more balanced at the cerebral level (LI = +0.11) than at the cerebellar level (LI = +0.66). Left and right are as indicated in the figure

The fMRI experiment was performed using a block-designed paradigm consisting of two conditions, each lasting for 30s: a resting period (R) and a noun-verb association task (T). Both these conditions were presented sixteen times in an alternating fashion: RT1-RT2-…-RT16. During the resting condition, no stimulus whatsoever was presented. In contrast, during the task of interest, a series of ten different Dutch nouns was presented with a 3 s interval. Different nouns were used in every block, 160 high frequent nouns were presented in total. A silent semantic noun-verb association task was used in which the patient had to think of a verb semantically related to a visually presented noun (as described in [42]). The axial and coronal slices of the fMRI are shown in Fig. 3.

A bilateral distribution of activations was found. The lateralisation index (LI) was calculated based on the number of voxels activated in the standard language areas (Broca: inferior frontal gyrus (BA 44–45); Wernicke: superior and middle temporal gyrus (BA 21–22), supramarginal gyrus (BA 40), and angular gyrus (BA 39)) and their homologue counterparts in the right cerebral hemisphere, according to the formula LI = (L-R)/(L + R) [43, 44]. Left-sided laterality is associated with a positive LI, right-sided laterality with a negative LI. Bilateral language representation is reflected by a LI between −0,25 and +0,25 [45, 46]. In this patient the LI indicated a clear bilateral distribution (LI = +0,11).

At the cerebellar level, fMRI revealed a clear left lateralised activation (LI = +0,66) with almost five times as many voxels activated in the left posterior cerebellar hemisphere than in the right posterior hemisphere (L: 1551 voxels; R: 318 voxels). Possibly this left lateralisation might be due to a loss of activation caused by damage to the right cerebellar hemisphere.

Bilateral hemispheric language representation was also suggested by DTI. DTI showed a slightly more pronounced arcuate fascicle on the right (231 tracts) than on the left (160 tracts), no significant difference was found in FA (R 0.4638; L 0.4429) or MD (R 0.000823 mm^2^/s; L 0.000827 mm^2^/s). The minimal difference between the number of tracts, the FA, and the MD also suggests a bilateral organisation of language functions at the cerebral level.

## Discussion

A wealth of studies has demonstrated that the cerebellum performs a crucial role in the modulation of a range of cognitive and affective functions via a dense network of crossed cerebello-cerebral connections [6, 9, 14, 15, 47, 48]. An increasing amount of evidence shows that the right cerebellar hemisphere modulates linguistic processes while the left cerebellar hemisphere is involved in typically nondominant hemisphere functions [49]. In this study, an infarction in the right posterior cerebellar hemisphere in a strongly right-handed patient caused a complex of frontal-like executive (impaired frontal problem solving and mental flexibility), behavioural (desinhibition and impulsivity), and attentional deficits (speed) consistent with Schmahmann’s syndrome. The remote functional impact of the cerebellar lesion on the prefrontal regions was confirmed by quantified SPECT demonstrating bilateral perfusional deficits in both frontal lobes. This is in accordance with the hypothesis that the cerebellum is involved in executive control via crossed cerebello-cerebral connections with the frontal association areas [11, 50].

It has been shown that the anatomo-functional language network is larger than previously assumed and includes regions at both the cerebellar and cerebral level. First, it is hypothesised that in most right- and left-handed subjects a right cerebellar lesion might induce language deficits following depression of function in the contralateral supratentorial language areas of the language dominant left hemisphere [19]. Mariën et al. (2007) [50] indeed identified linguistic deficits in their patient after a right cerebellar lesion due to decreased perfusion in the left prefrontal language regions. In this patient, however, language deficits were ruled out by a number of formal test batteries. Second, it has been shown that the left insular region is of vital importance for articulated speech [2] and language [3, 4, 51, 52]. However, in this patient an old vascular lesion in the left anterior insular region extending to the prefrontal gyrus was never associated with any motor speech (planning) or language disturbances. Both the right cerebellar hemisphere and the left insular region are critically implicated in language processing and there also seems to exist a close functional interplay between both areas [19]. Mariën et al. (2001) [19] hypothesised that due to the striking semiological similarities between apraxia of speech (caused by lesions in the left anterior insula) and ataxic dysarthria (caused by lesions in the right cerebellum), both areas are anatomically and functionally interconnected to subserve language functions [19]. It is therefore remarkable that this patient never presented with any speech or language problems.

To explore this apparent contradiction, fMRI was performed which showed bilateral cerebral activations during a silent semantic association task. The LI of this patient (= +0,11), based on the activation in the left language areas and their homologue right counterparts, demonstrated a clear bilateral organisation of language in the cerebral hemispheres (−0,25 ≤ LI ≤ 0,25) [45, 46]. In normal healthy adults, this task only activates the frontal opercular cortex, the insular cortex, and a small region in the posterior superior temporal cortex bilaterally [53]. In this patient, the left cluster in the superior temporal cortex was activated to a slightly greater extent in the homologue right region. The frontal opercular cortex on the other hand was only activated on the left side. Surprisingly, no insular activation was found, left nor right. On the left, this is probably due to the old insular infarct. To explain the lack of activation on the right it might be hypothesised that in addition to an atypical distribution of language functions at the interhemispheric level, an atypical intrahemispheric organisation might be expected. An anomalous intrahemispheric organisation of language functions is frequently observed in atypical populations such as aphasia patients with crossed language dominance. In crossed aphasia, anomalous anatomoclinical configurations at the intrahemispheric level are found in 39% of the population [21, 54].

DTI, in addition, showed equal sized arcuate fascicles in the right and the left hemisphere, which might indicate long-standing bilateral language representation. The arcuate fascicle connects Broca’s area with Wernicke’s area and plays an important role in language [55]. Although a denser (higher fiber density) arcuate fascicle on the left does not always indicate left-sided language dominance, it has been shown that in right-handers the degree of structural asymmetry correlates with the degree of functional lateralisation [56]. An equal sized arcuate fascicle in the right and left hemisphere is therefore a strong indicator of a bilateral language representation.

Since linguistic functions in subjects with atypical bilateral language representation are less dependent on one language dominant hemisphere, it might not be surprising that no language disturbances were detected after left insular and right cerebellar damage. It could be argued that in patients with a bilateral language organisation, the contralateral homologue regions can flawlessly and apparently instantly compensate for possible functional loss due to cerebral or cerebellar damage. In this study, fMRI confirmed a much stronger activation of the left cerebellar hemisphere during the semantic noun-verb association task (LI = +0,66), despite an evenly distributed activation at the cerebral level. Although it could be argued that the asymmetry was caused by the right cerebellar lesion, this would imply a loss of linguistic function due to loss of activation. Since no linguistic deficits were observed, it is reasonable to assume that either (1) there was no loss of activation and the language functions were always strongly left lateralised in the cerebellum, or (2) the loss of function due to cerebellar damage is compensated by a stronger activation of the left cerebellum. A recent study by Mendez Orellana et al. (2015) [57] showed a significant dependency between cerebral and cerebellar language lateralisation both in tumour patients and in healthy subjects, which was almost always crossed. This study indicated that a left cerebellar language dominance is strongly associated with a right hemisphere language dominance at the supratentorial level. Thus, if left language lateralisation was present before the patient sustained acute right cerebellar damage, this would suggest a right lateralisation at the cerebral level. However, fMRI results contradict a right hemisphere language dominance, and together with DTI a congenital bilateral language distribution is far more likely, which, according to the study of Mendez Orellana et al. (2015) [57] is associated with either a symmetrical (bilateral) activation at the cerebellar level or with a right cerebellar lateralisation, excluding the first hypothesis. The unusual lateralisation of language function in the left cerebellar hemisphere might indicate that the left cerebellum compensated the functional loss in the right cerebellum after acute damage in a context of a bilateral organisation of language function, as was put forward in the second hypothesis. However, to confirm this hypothesis a more systematical analysis of subjects with congenital bilateral organisation of language functions should be performed.

## Conclusion

This exceptional patient developed no language deficits after a left insular and a right cerebellar stroke. By contrast, Schmahmann’s syndrome was identified after the cerebellar infarct and reflected on SPECT by crossed cerebello-cerebral diaschisis in the anatomoclinically suspected frontal regions. Evidence from fMRI and DTI in this patient suggests atypical bilateral language organisation as a maturational variant of cerebral language representation. Possibly, a bilateral distribution of language allows for a better and faster compensation of functional loss due to acute cerebral or cerebellar damage.

## Consent

Written informed consent was obtained from the patient for publication of this case report and any accompanying images. A copy of the written consent is available for review by the Editor-in-Chief of this journal.
